# The Institutional Library

**Published:** 1919-05-03

**Authors:** 


### THE INSTITUTIONAL LIBRARY.

### WAR NEUROSES.

THE medical and surgical experiences of the war will in due course form an important library, and the lessons they have taught will be summarised for future guidance. There is just now a widespread hope that no similar occasion for the application of these lessons will arrive, but no one can feel quite sure, and the prudent man resolves to keep his powder dry. In any event, the teaching has many points of contact with medical practice in civil life, and here certainly its opportunity for influence will not fail. Among other instances this influence will be seen, somewhat unexpectedly perhaps, in the field of functional nervous disease. For long prior to the war physicians were familiar with cases where under various stresses the individual showed himself incapable of meeting the demands of the situation in which he was placed, and various hypotheses were advanced to explain this condition and to afford a guide to successful treatment. The patient was not the subject of organic disease; he was not a malingerer; he was not insane. All the same, he was ill in the sense that his capacity for usefulness or for enjoyment was for the time being in suspense. Popularly he was described as the victim of a "nervous breakdown," and a more technical diagnosis would dub him hysterical or neurasthenic or hypochondriachal. It was to cases of this order that the process of psycho-analysis became applied, and the method evoked a very vigorous controversy, largely because of the sex associations which some of the leaders of the school affirmed as the basis of all such disturbances. With the war there came obviously a supreme test of the capacity of men to adapt themselves to conditions in which new and severe stresses took a prominent place. Little wonder, therefore, that in thousands of instances failures appeared, and that many men who could meet, and had met successfully, the demands of civilian life were unable to withstand the mental strain and sustained anxiety of an active campaign. The opportunities for the study of such cases were in this way increased on an enormous scale, and as the literature dealing with what has been loosely termed "shell-shock" shows, these opportunities excited great interest.

The book now before us* is one of many dealing with "war neuroses" on the basis of a considerable personal experience, and in our judgment it must be placed in the first rank. The author came to this country in 1917 for the express purpose of studying a problem which, in consequence of the entrance of America into the war, his fellow-countrymen would shortly have to face. He was well fitted for the task both by natural aptitude and by acquired knowledge and experience. If any justification were needed for his appointment it is found in the present volume. The investigation of psycho-neuroses has made little appeal to the general body of the profession, and, as has already been indicated, the enterprise has been looked at somewhat askance. This attitude will have to be revised, and the changed outlook will exert its influence on civil practice. It is impossible to read Dr. MacCurdy's pages and to escape the conviction that a new hopefulness is introduced into the scientific definition of many mental and emotional disturbances and also into their successful treatment, and this is the more welcome seeing that by the majority of the medical profession such cases were regarded with impatience or with despair. We wish to emphasise the general field of application of these studies, because now that the war is over the title "War Neuroses" may seem to deal with a state of matters whose interest has largely evaporated. Such a conclusion is quite unwarranted. Whether considered as an example of scientific investigation or as a therapeutic contribution to a difficult branch of practical medicine, Dr. MacCurdy's book should be read by every practitioner. It has a high educational value, and its teaching affords helpful guidance in difficult and not unfamiliar situations. Not the least of its attractions is a literary style that leaves nothing to be desired, and it may indeed be quoted as an example of the fashion in which scientific thoroughness may be associated with clear thought and simple and lucid speech. Even the non-technical reader may enjoy its perusal unburdened by the fear of a polysyllabic vocabulary.

The book, in a word, may be described as a study in natural history, and its subject is the attempt of modern men to adapt themselves to the conditions of modern warfare. Why some succeed in this attempt and others fail is a matter for scientific inquiry. The failures must be analysed, and from the analysis emerges a classification. To the uneducated eye there appears an almost infinite number of disturbances, but Dr. MacCurdy finds that all fall into one or other of two classes. The one group he names "Anxiety States" and the other "Conversion Hysterias." In the first are symptoms having anxiety as their basis; in the second a morbid psychical energy has been converted into obvious physical disturbances. The development of one variety or the other is largely dependent on the nature of the desires entertained by the soldier prior to his breakdown. As Dr. Rivers puts it in his helpful Preface, one of the most original features of Dr. MacCurdy's book is a recognition of the truth that those who suffer from Anxiety States have wished for death during the period of strain and fatigue preceding the final collapse, while sufferers from Conversion Hysterias have entertained the desire for disablement. How these conclusions have been reached must be sought in the book itself, and we can only repeat that every one who elects to read it has in front of him an enjoyable and an educative experience.

* By John T. MacCurdy, M.D. With a Preface by W. H. R Rivers, M.D. (Cambridge: The University Press. 1918. Pp. 132 + ix. Price 7s. 6d. net.)

### Sir William Ramsay as a Scientist and Man.*

THIS small volume is a tribute to the life and work of the late Sir William Ramsay, and it is written by two Professors of Chemistry in Government colleges in India. Some fifteen pages of it are monopolised by a list of Ramsay's contributions to various scientific journals. The rest of the book is occupied by an appreciation of his achievements as an investigator and of his excellent personal qualities. The whole testifies to the goodwill of the authors and to their admiration for a man whose work is certain of an abiding place in the science to which he devoted his life. For the general reader the matter is somewhat severely technical, while the expert will be inclined to desire a more detailed and critical examination. But the motive of the book is excellent and is well deserving of sympathy.

* By Tarini Charan Chaudhuri, M.A. With an introduction by Panchanan Neogi, M.A., Ph.D. (Calcutta: Butterworth and Co., Ltd. 1918. Pp. 66 + ix. Price R. 1.8.)

**The Institutional Library**—(*continued*).

### The Military Medical Manuals.

(London: The University of London Press, 18 Warwick Square, London, E.C. 4. Price 7s. 6d. each volume.)

The admirable series of volumes of some 250 pages each covers the whole field of war experiences in both medicine and surgery, and each one gives as up-to-date a review of the particular sections considered as we have yet seen in book form. Either as a set whereby the general practitioner can keep himself abreast of the trend of modern medical thought, or as individual volumes from which the specialist can derive most useful information concerning his favourite branch of the profession, these volumes meet with our unqualified approval. Below we refer to two of them.

**I. Mental Disorders of the War.** By Professor Jean Lepine, of the University of Lyons, and edited by Charles A. Mercier, M.D., F.R.C.P., F.R.C.S.

The intention has here been to assist those medical men who have no previous specialised study or experience of neuro-psychiatry to deal with this particular type of case in any of the various phases of military environment where they are met. Classification and depth of detail is guided mainly by military rather than the classical consideration of mental disease. Description is first made of clinically acute cases, followed by those which appear as chronic from the onset, and then attention is directed to certain special types. A medico-legal survey of the functions of an expert in relation to questions of responsibility, and a consideration of decisions between which the doctors and the commissioners have to choose occupy the final chapters. The editor is to be congratulated upon a graceful translation, in which he has entirely preserved the spirit as well as literal facts of the French author's work.

**II. Electro-Diagnosis in War.** By A. Zimmern, Professeur Agrégé of the Faculty of Paris, and Pierre Perol, formerly Intern of the Hospitals of Paris. Edited by E. P. Cumberbatch, M.A., M.R.C.P.

From this volume one derives much practical information as to the technique and application of electrical reactions, not in war conditions alone, but in every case of nerve lesion. The plea is for greater care and thoroughness in testing, and all causes of failure are fully reviewed. The greatest advantage lies in the fact that objective signs are obtained in which the personal element plays no part, and as such should interest all concerned in the work of medical boards. The subject is highly technical, and not easy to follow at the outset; but with the aid of excellent diagrams and simple, straightforward explanation of clinical details, the authors have provided at once a most useful book of reference and a concise account of a definite scientific advance.

**The Adventure of Life.** By Robert W. MacKenna, M.A., M.D. (London: John Murray, 1919. Price 6*s.* net.)

This is one of the many books which have been, and will continue to be, written in the attempt to explain in popular form the origin and purpose of life. In a fluency of language and an agreeable bustle of mind, Dr. MacKenna casts his net wide over the waters, and discusses in a series of chapters the origin of life, the evolution of man, free will, pain, marriage, death, and "the revelation of God to man." Much of it is necessarily a summary of theories, and as such it cannot be reviewed, for almost every statement is inevitably open to dispute. The author is strongest, perhaps, on the scientific side, and the layman who likes this kind of book will enjoy the procession of facts and theories which are hurried rapidly before him. A breezy optimism pervades the whole, which seeks to support the writer's conclusion that "the goal of Nature is life; the aim of life is the development of intelligence, and the object of intelligence is a knowledge of God." Its reading is rather a breathless business, and we finish the book feeling that if less had been attempted more might have been gained. Dr. MacKenna has been well served by his publishers, who have given him paper and binding beside which many books now being issued at the same price are put to shame.

**Dr. Elsie Inglis.** By Lady Francis Balfour, with illustrations. (Hodder & Stoughton. Price 6*s.* net.)

A very notable woman lives in the pages of this brief biography. Dr. Inglis played a conspicuous part in the battle for the recognition of female medical students, and would have won a place in the history of surgery even had her later adventures not pushed her into fame. Many readers will linger over the chapters recounting her early difficulties and her struggles to open the gate of opportunity to her students when she had forced it open for herself. They are the best in the book. The Serbian expedition is hardly so well encountered. The material to hand is not too well assimilated, and without some previous knowledge of the course of events is almost unintelligible. For instance, the epitaph and tributes to Dr. Inglis's memory after she died, contributed by Serbians, appear in the chapter which recounts the events of 1915. The letters interpolated miss their effect for lack of any coherent account of what actually happened, and a great opportunity for writing a noble page of history has in consequence been thrown away. The book contains valuable material. But the definitive life of Elsie Inglis has yet to be written.

**The Doings of Donovan In and Out of Hospital.** Pictured by J. H. Dowd, with a Foreword by W. Pett Ridge. (Published by Country Life, Ltd. Price 3s. 6d. net.)

These drawings by Mr. Dowd are already well known to our readers from the regular reviews which we have published of the *Gazette* of the 3rd London General Hospital, Wandsworth, in which they first made their appearance, from the article which we published in the "Artists of the 3rd London" when they held an exhibition at the Camera Club a year ago, and from certain reproductions which have been made from them in The Hospital. Their separate publication should reach a wider audience, and doubtless will also come as a pleasant aid to those whose files of the original issues of the *Gazette* are incomplete. The rough-and-tumble humour of the drawings needs no emphasis again here, nor the occasional flashes of wit which dignify them. Their relation to the real Tommy is put well and tersely by Mr. Noel Irving in a brief introduction which is an ornament to the book.

